# Mechanosensitivity of phase separation in an elastic gel

**DOI:** 10.1140/epje/s10189-024-00405-y

**Published:** 2024-02-20

**Authors:** Dan Deviri, Samuel A. Safran

**Affiliations:** 1 Department of Chemical and Biological Physics, Weizmann Institute of Science, 76100 Rehovot, Israel; 2 Carbon Blue Ltd., 3303201 Haifa, Israel

## Abstract

**Abstract:**

Liquid–liquid phase separation (LLPS) in binary or multi-component solutions is a well-studied subject in soft matter with extensive applications in biological systems. In recent years, several experimental studies focused on LLPS of solutes in hydrated gels, where the formation of coexisting domains induces elastic deformations within the gel. While the experimental studies report unique physical characteristics of these systems, such as sensitivity to mechanical forces and stabilization of multiple, periodic phase-separated domains, the theoretical understanding of such systems and the role of long-range interactions have not emphasized the nonlinear nature of the equilibrium binodal for strong segregation of the solute. In this paper, we formulate a generic, mean-field theory of a hydrated gel in the presence of an additional solute which changes the elastic properties of the gel. We derive equations for the equilibrium binodal of the phase separation of the solvent and solute and show that the deformations induced by the solute can result in effective long-range interactions between phase-separating solutes that can either enhance or, in the case of externally applied pressure, suppress phase separation of the solute relative to the case where there is no gel. This causes the coexisting concentrations at the binodal to depend on the system-wide average concentration, in contrast to the situation for phase separation in the absence of the gel.

**Graphical abstract:**

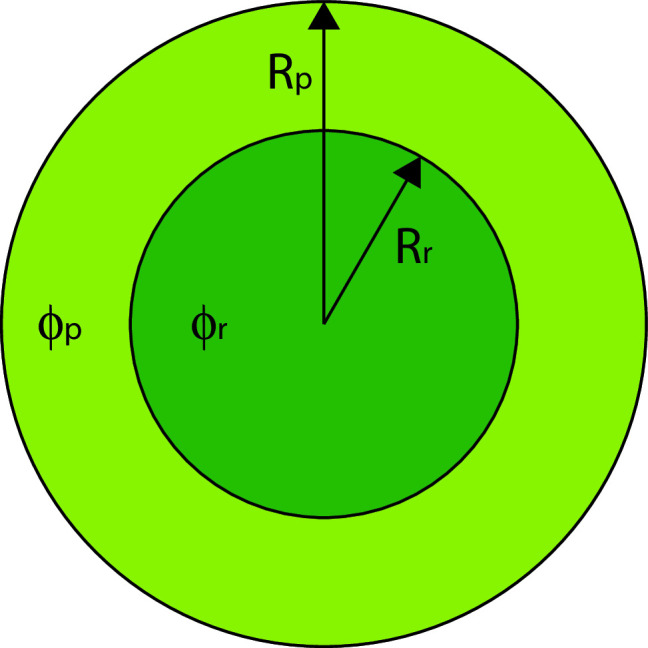

**Supplementary Information:**

The online version contains supplementary material available at 10.1140/epje/s10189-024-00405-y.

## Introduction

Liquid–liquid phase separation (LLPS) of solutes in a solvent occurs when short-range attractive interactions, enthalpic or entropic, dominate the mixing entropy that tends to homogenize the solutes. Phase separation phenomenon is ubiquitous in nature across many scales and is responsible for the self-assembly of supra-structures ranging from clouds [1] to subcellular organelles [2] and fractal materials [3]. In the past, theoretical studies of LLPS have focused on the case where the attractive interactions between the phase-separating solute particles are short-ranged. Long-range interactions were studied primarily in the context of metallurgy and hydrogen embrittlement, where the elastic stress fields induced by inclusions (hydrogen atoms) lead to long-range attraction and phase separation of the inclusions, which then lead to embrittlement of the metal [4].

The renaissance of soft matter physics over the past decades highlights many self-assembling elastic systems, such as gels and membranes, in which soluble molecules and macromolecules modulate the mechanical properties of the systems [5, 6]. This raises the interesting question of the interplay between the phase separation behavior of the soluble molecules and the long-range elastic interactions induced by the phase separation of these molecules due to the elastic medium surrounding the phase-separated regions. Such long-range interactions were recently studied in the context of polymer–polymer phase separation, where a gel immersed in a liquid undergoes phase separation due to attractive interactions of its polymer constituents [7]. For a gel hydrated by a phase-separating solution, a limiting case of these interactions, in which the phase-separated body completely excludes a gel surrounding it, was studied theoretically and experimentally in in-vitro soft matter systems [8–10] and in-vivo inside the cell nucleus [11, 12]. However, a theoretical understanding of the opposite case, in which a gel permeates both phase-separated domains [13], is lacking.

In this paper, we formulate and solve a theoretical model for such a system comprised of an elastic gel hydrated by an aqueous solution of water and phase-separating molecules that interact with the gel and modulate its mechanical properties. For simplicity of the model, we limit the effect of the phase-separating molecules on the gel to modulation of its bulk modulus but not its shear modulus. As explained in the Model and Results section below, while our simplified model is generic, it is inspired by the case of polyelectrolyte gels [14] immersed in a solution of phase-separating, oppositely charged molecules. Our model predicts that the long-range elastic interactions of the gel lead to unique phase separation properties, such as modulation of phase separation by external elastic stresses. In addition, the solute concentrations in the coexisting phase-separated domains depend on the average, system-wide solute concentration. This differs from phase separation in the absence of long-range elastic interactions, where the solute concentrations in the two coexisting phases depend only on the magnitude of the short-ranged attractive interactions and temperature and not on the overall (system-averaged) solute concentration. This is inherent in the nature of the tie lines and in the fact that the lever rule [15] predicts only the macroscopic volumes of the two coexisting domains. The fact that the thermodynamic forces result in well-defined coexisting concentrations, independent of the overall concentration, means that LLPS systems prepared with a variety of overall concentrations will all show the same two coexisting concentrations. This is known as “concentration buffering” in biological systems where the protein concentration in aqueous solution is subject to noisy gene expression [16].

An important consequence of our theoretical predictions may be their implications for the crosstalk of the mechanical (gel-like) environment and protein solutes in biological LLPS. For example, the involvement of some nuclear phase-separated bodies, termed transcriptional condensates, in control over gene expression is well documented [17]. Interestingly, the results of our model suggest a novel physiological function of such condensates. Both in-vitro and in-vivo, live organism experiments indicate that chromatin [18] is condensed at the nuclear scale due to effective self-attraction [19–22]. In the case of strong attractions or actual cross-linking by chromatin binding proteins, the chromatin can act as a gel, at least within some time scale. If the molecules comprising such condensate also change the mechanical properties of the chromatin gel, the entire transcriptional condensate may result in emergent mechanosensitivity; namely, the condensate can convert mechanical signals to downstream changes in the gene expression without requiring the proteins comprising the condensate to be mechanosensitive.

## Model and results

In the spirit of the theoretical work of Onucki on spinodal decompositions of alloys and gels [23, 24], we use a similar free energy density. This free energy includes both the solute free energy (mixing entropy and attraction between the solutes that leads to phase separation) and the elastic interaction within the surroundings which is a gel in both the one-phase and phase-separated states of the solute and solvent.

1 $$\begin{aligned} f= & {} f_\textrm{s}\left( \phi \right) +\alpha \phi u_{\ell \ell }+\frac{\lambda _\textrm{r}\left( \phi \right) }{2}u_{\ell \ell }^{2}+\mu _\textrm{r}\left( \phi \right) u_{ik} u_{ik} \end{aligned}$$

where *f* is the free energy density, $\phi$ is the volume fraction of the solute, $f_\textrm{s}\left( \phi \right)$ is the free energy density of the solute, and $u_{ij}$ are the components of the strain tensor (where one sums over repeated indices). The quantities $\lambda _\textrm{r}\left( \phi \right)$ and $\mu _\textrm{r}\left( \phi \right)$ are, respectively, the renormalized first and second Lamé coefficients of the elastic medium, which depend on the solute concentration, $\phi$, while $\alpha$ is a phenomenological constant related to the expansion or shrinking of the surrounding elastic medium by the solute molecules. We note that the polymers comprising the gel are incompressible at the molecular level and the Lamé constant and bulk modulus (as defined below) refer to the densification of the gel due to its hydration by the solvent (and solute). This free energy was originally formulated to investigate spinodal decomposition in alloys and gels. We focus on the case where fraction of the gel polymer itself is negligible compared to that of the solvent, and the mesh size, $\xi$, of the gel is much larger than the size of a solute molecule. In such, relatively dilute gels, the effect of the gel on the solution-free energy density $f_\textrm{s}$ is negligible. This is because the elastic energy per unit volume of the gel scales as $k_\textrm{B}T/\xi ^{3}$, while the free energy density of the solute scales as $k_\textrm{B}T/a^{3}$, where *a* is the molecular size, assumed to be much smaller than the mesh size. This is appropriate away from the critical point of the phase separation and we limit our discussion to the regime of strong segregation (where the solution correlation length is much smaller than the mesh size. In this case, $f_\textrm{s}$ depends only on $\phi$ and involves the solute entropy and its short-ranged interactions with the solvent, but not gel. Hence, $f_\textrm{s}$ is not a function of strain. However, as we explain below, the effect of the solute on the mechanical properties of the gel is not necessarily negligible.

Our free energy is generic, but can be motivated by the example of a polyelectrolyte biogel, such as (net) negatively charged [18] chromatin in aqueous solution. In the well-screened (high salt concentration) regime, the osmotic pressure of the counterions localized to the volume of the chromatin to neutralize its charge provide major contribution to the bulk modulus of the gel [25]. However, the presence of positively charged proteins in the solution permeating the chromatin may allow the release of counterion pairs outside of the gel volume [26], thereby reducing the bulk modulus of the gel. If these proteins are also cross-linkers of the chromatin gel, they can also modulate the shear modulus of the gel [27]; conversely, if the proteins have the same charge as the chromatin the bulk modulus of the gel increases. In what follows, we discuss the generic case which depends only the short-ranged, solute–solvent interactions and their effect on the gel modeled as a linearly elastic network.

For simplicity of the model, we limit ourselves to the case where the solute only affects the bulk modulus and not the shear modulus, so that $\mu _\textrm{r}\left( \phi \right) =\mu$, which we now relate to a molecular picture of polyelectrolyte gels that can motivate our free energy. The bulk modulus is defined phenomenologically as the coefficient of linear stress response of an elastic material to deformations that change its volume but not shape (i.e., a uniform expansion or contraction that does not change the relative positions of its molecular constituents). Conversely, the shear modulus is the linear stress response to deformations that change the shape, but not the volume of the elastic material. As explained above, the osmotic pressure of counterions is a primary contributor to the response of electrolyte gels to changes in their volume by compression or expansion. Therefore, any solute that affects the concentration of counterions, and thus their osmotic pressure, will modulate the bulk modulus of the gel. Also as explained above, charged solutes that bind the gel polymers may change counterion concentration and, consequently, the osmotic pressure and bulk modulus of the gel. In contrast to the bulk modulus, the shear modulus is indifferent to the presence of counterions, which in the well-screened regime behave as an ideal solution that can change shape without any energy cost. In the context of gels (polyelectrolyte or otherwise), cross-linkers are the dominant determinants of the shear modulus [28] because they resist shape changes of the network formed by the polymers. Therefore, the solutes we consider in this work, which affect the bulk modulus but not the shear modulus, are not cross-linkers; namely, the solutes bind the polymer constituents of the gel in only one site (monovalent) and not two or more (multivalent) that may create cross-links. We further simplify the model by considering only the linear dependence of the modulated first Lamé coefficient on $\phi$, $\lambda _\textrm{r}\left( \phi \right) =\lambda +\beta \phi$, where $\beta$ is a phenomenological constant; $\lambda$ and $\mu$ are Lamé coefficients of the gel in the absence of the solute; they are thus independent of $\phi$. Substituting these relations into Eq. 1 results in the following model-specific free energy:

2 $$\begin{aligned} f=f_\textrm{s}\left( \phi \right) +\alpha \phi u_{\ell \ell }+\frac{\lambda }{2}u_{\ell \ell }^{2}+\frac{\beta }{2}\phi u_{\ell \ell }^{2}+\mu u_{ik} u_{ik} \end{aligned}$$

As in any other elastic system, force balance dictates that the divergence of the stress tensor vanishes in mechanical equilibrium. However, in our system, the mechanical properties of the gel are coupled to the local concentration of the solute, which implies that the stress tensor is also $\phi$-dependent. We calculate the stress tensor of our system, $\sigma _{ij}$, from the functional derivative [29] of the free energy *F* (which is the volume integral of Eq. 2) taken with respect to the strain tensor. We then find the divergence of the stress tensor and write the equation describing the mechanical equilibrium of the system:

3 $$\begin{aligned} \sigma _{ij}= & {} \frac{\delta F}{\delta u_{ij}}=\alpha \phi \delta _{ij}+\left( \lambda +\beta \phi \right) u_{\ell \ell }\delta _{ij}+2\mu u_{ij} \end{aligned}$$

4 $$\begin{aligned} \nabla \cdot \sigma= & {} \nabla \left( \left( \lambda +\mu +\beta \phi \right) \left( \nabla \cdot \vec {u}\right) +\alpha \phi \right) +\mu \nabla ^{2}\vec {u}=0\nonumber \\ \end{aligned}$$

where $\vec {u}$ is the displacement vector of the system.

**Fig. 1:**
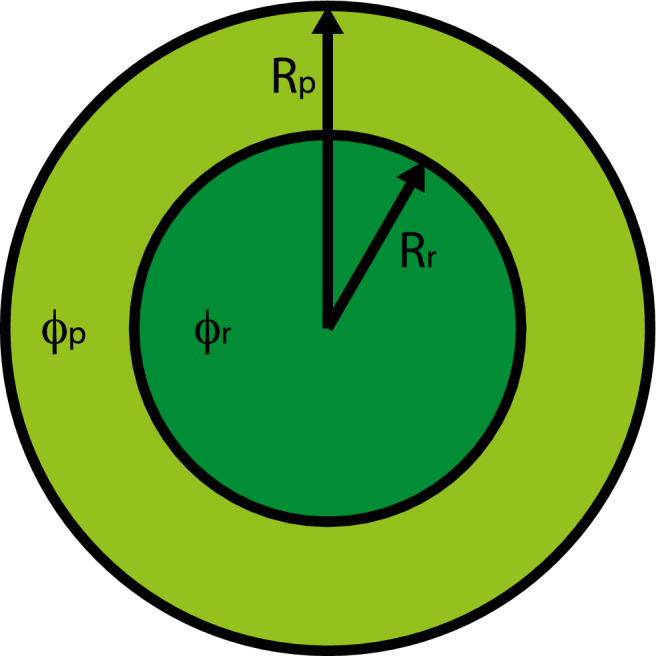
A schematic cartoon of the spherically symmetric model of a phase-separating solution in a gel. The overall radius of the gel is $R_\textrm{p}$, and the solute-rich domain that is formed by phase separation is a concentric sphere of radius $R_\textrm{r}$. The concentration of the solute in the solute-rich phase (dark green) is $\phi _\textrm{r}$, and in the solute-poor phase (light green) is $\phi _\textrm{p}$

Motivated by the cell nucleus, in which the chromatin behaves as a polyelectrolyte gel [6, 22] that is tethered to a spherical shell of lamina which defines the radius of the nucleus [30], we consider a spherical gel of radius $R_\textrm{p}$. We further simplify the system by considering the spherically symmetric case: one phase-separated domain is a sphere of radius $R_\textrm{r}$, concentric with the outer boundary of the gel of radius $R_\textrm{p}>R_\textrm{r}$) that contains the other phase-separated domain (see Fig. 1). For this symmetry, the displacement vector of the gel is only a function of the radial coordinate *r*, and the solution of Eq. 4 is simply.

5 $$\begin{aligned} \vec {u}=\left( \frac{A}{3}r+\frac{B}{r^{2}}\right) \hat{r} \end{aligned}$$

where *A* and *B* are integration constants determined by the boundary conditions of the system.

We now use the model described above to investigate the mean-field, equilibrium, binodal curve of the gel–solvent–solute system away from the critical point. In the two-phase regime, the binodal is the locus of points that delineate (at each temperature and pressure) the concentrations of the two coexisting phases. To predict those concentrations, we assume that the system is phase-separated, treat the relative volumes of the coexisting phase and their solute concentrations as unknowns, and write the total free energy of the system as a function of these unknowns. We next determine the binodal curve. We minimize the free energy under the constraints of constant chemical potential and constant osmotic pressure [31]. Notably, the solute concentrations within the two coexisting domains are constant, which, as we now explain, is related to the requirement of equal chemical potential of the solute in each of the two phases. Differentiation of the free energy in Eq. 2 results in the following expression for the chemical potential, $\mu =f'_\textrm{s}\left( \phi \right) +\alpha u_{\ell \ell }+\beta u_{\ell \ell }^{2}/2$. Substitution of Eq. into the expression for $u_{\ell \ell }$ results in $u_{\ell \ell }=A$ which in turn implies that $f'_\textrm{s}\left( \phi \right) =\mu -\alpha A-\beta A^{2}/2$ which is a constant within each of the coexisting domains. Thus, the solute concentration $\phi$ must also be a constant. We denote the two coexisting concentrations of the solute by $\phi _\textrm{r}$ and $\phi _\textrm{p}$ for the solute-rich and solute-poor phases, respectively. The interface between the two phases is denoted by the radius $R_\textrm{r}$, which determines the relative volumes of the two domains. Since the typical length scale of an interface between two phase-separated domains, which are away from the critical point, is of the order of the molecular size, which is smaller than the typical mesh size of a gel, we neglect the interface width. Lastly, for convenience, we choose the solute-rich phase to be in the center of the sphere; we show below that this choice is arbitrary and does not change the free energy.

We now write the equations for the two pairs of integration constants that characterize the displacement vector in the two coexisting domains: $A_\textrm{r}$ and $B_\textrm{r}$ for the inner, solute-rich phase and $A_\textrm{p}$ and $B_\textrm{p}$ for the outer, solute-poor phase. These equations represent four conditions: (1) The displacement cannot diverge, implying that $B_\textrm{r}=0$; (2) the displacements must be equal across the interface, $\vec {u}\left( R_\textrm{r}^{+}\right) =\vec {u}\left( R_\textrm{r}^{-}\right)$, where $+$ and −, respectively, denote infinitesimal distances greater and less than $R_\textrm{r}$; (3) the normal stress must be equal across the interface (mechanical equilibrium), $\sigma _{rr}\left( R_\textrm{r}^{+}\right) =\sigma _{rr}\left( R_\textrm{r}^{-}\right)$ and; (4) the stress or displacement fixed by a boundary condition at the surface of the gel at $r=R_\textrm{p}$. In this work, we consider the boundary condition of constant stress at the surface of the sphere; the boundary condition of zero displacement at the surface of the spherical gel is solved in the SI.

For the constant stress boundary condition, the stress at the surface of the spherical gel is constant and equal to the externally imposed stress. To keep the system spherically symmetric, we consider a stress that originates in a constant hydrostatic pressure *p*, so that $\sigma _{rr}\left( r=R_\textrm{p}\right) =p$. This condition, together with no-divergence and continuity conditions of the displacement and mechanical equilibrium, forms a system of four equations and four variables (the integration constants) whose solution leads to the following expression of the displacement (see SI):

6 $$\begin{aligned} \vec {u}= & {} \hat{r}\cdot \left\{ \begin{array}{cc} \frac{1}{3}\frac{K_\textrm{p}\left( p-\alpha \phi _\textrm{r}\right) +\frac{4\mu }{3}\left( p-\alpha \bar{\phi }\right) }{K_\textrm{r}K_\textrm{p}+\frac{4\mu }{3}\bar{K}}r &{} r<R_\textrm{r}\\ \frac{r}{3}\frac{K_\textrm{r}\left( p-\alpha \phi _\textrm{p}\right) +\frac{4\mu }{3}\left( p-\alpha \bar{\phi }\right) }{K_\textrm{r}K_\textrm{p}+\frac{4\mu }{3}\bar{K}}-\frac{1}{3}\frac{\left( \alpha K+\beta p\right) \left( \phi _\textrm{r}-\phi _\textrm{p}\right) }{K_\textrm{r}K_\textrm{p}+\frac{4\mu }{3}\bar{K}}\frac{R_\textrm{r}^{3}}{r^{2}} &{} R_\textrm{r}<r<R_\textrm{p} \end{array}\right. \end{aligned}$$

where $K=\lambda +2\mu /3$ is the bulk modulus of the gel, $\bar{\phi }=\left( V_\textrm{r}\phi _\textrm{r}+V_\textrm{p}\phi _\textrm{p}\right) /V$ is the system-wide average solute concentration, and $K_\textrm{r}=K+\beta \phi _\textrm{r}$, $K_\textrm{p}=K+\beta \phi _\textrm{p}$, and $\bar{K}=K+\beta \bar{\phi }$ are the effective bulk moduli in solute-rich phase, solute-poor phase, and the single phase that preceded phase separation, respectively; $V_\textrm{r}=4\pi R_\textrm{r}^{3}/3$ and $V_\textrm{p}=4\pi \left( R_\textrm{p}^{3}-R_\textrm{r}^{3}\right) /3$ are, respectively, the volumes of the solute-rich and solute-poor phases, and $V=V_\textrm{r}+V_\textrm{p}=4\pi R_\textrm{p}^{3}/3$ is the total volume of the spherical gel. $\bar{\phi }$ is also the average solute concentration in the one-phase region, so one does not have to specify the volumes of each of the phase-separated domains to determine $\bar{\phi }$.

We now find the total free energy of the system as a function of the volumes of the two phases and the coexisting concentrations by deriving the strain tensor from the displacement of Eq. 6 and substituting it into the expression of the free energy density in Eq. 2. Then, integrating the free energy density over the volume of the spherical gel results in the total free energy *F* of the system (see SI).

The free energy includes the contribution of the solutes of the two phases, identical to the free energy of a liquid system undergoing LLPS. However, there are additional terms in the free energy that account for the contribution of the long-range elastic interactions. Since the elasticity is long-range these terms couple the concentrations and volumes of the two phases, the free energy and, as we now show, the criteria for coexistence of solute-rich and solute-poor phases involve the system-averaged concentration $\bar{\phi }$; this is in contrast to LLPS in the absence of the gel.

The free energy of the phase-separated system gives rise to a grand potential $G=F+\eta \left( V_\textrm{r}\phi _\textrm{r}+V_\textrm{p}\phi _\textrm{p}\right) +\pi \left( V_\textrm{r}+V_\textrm{p}\right)$ allowing us to minimize *G* subject to the conditions of equal chemical potentials and osmotic pressures in the two phases [31]. This minimization results in a pair of equations that determine the binodal (coexistence curve) of the system (see SI) and hence the concentrations $\phi _\textrm{p}$ and $\phi _\textrm{r}$ in the each phase.

7 $$\begin{aligned}{} & {} \frac{f_\textrm{s}\left( \phi _\textrm{r}\right) -f_\textrm{s}\left( \phi _\textrm{p}\right) }{\phi _\textrm{r}-\phi _\textrm{p}}\nonumber \\{} & {} \quad = \frac{\partial f_\textrm{s} \left( \phi _\textrm{r}\right) }{\partial \phi _\textrm{r}}-\frac{1}{2}\frac{\left( K^{2}\alpha ^{2}-p^{2}\beta ^{2}\right) \left( M+\beta \phi _\textrm{p}\right) \left( \phi _\textrm{r}-\phi _\textrm{p}\right) }{\left( K_\textrm{r}K_\textrm{p}+\frac{4\mu }{3}\bar{K}\right) ^{2}}\nonumber \\ \end{aligned}$$

8 $$\begin{aligned}{} & {} \frac{f_\textrm{s}\left( \phi _\textrm{r}\right) -f_\textrm{s}\left( \phi _\textrm{p}\right) }{\phi _\textrm{r}-\phi _\textrm{p}}\nonumber \\{} & {} \quad = \frac{\partial f_\textrm{s}\left( \phi _\textrm{p}\right) }{\partial \phi _\textrm{p}}+\frac{1}{2} \frac{\left( K^{2}\alpha ^{2}-p^{2}\beta ^{2}\right) \left( M+\beta \phi _\textrm{r}\right) \left( \phi _\textrm{r}-\phi _\textrm{p}\right) }{\left( K_\textrm{r}K_\textrm{p}+\frac{4\mu }{3}\bar{K}\right) ^{2}}\nonumber \\ \end{aligned}$$

where $M=K+4\mu /3$ is the p-wave modulus [32] of the gel, related to its bulk and shear moduli. The binodal equations (Eqs. 7 and 8) are symmetric to interchange of the coexisting concentrations of the phase-separated domains, $\phi _\textrm{r}\leftrightarrow \phi _\textrm{p}$, implying that the choice of positions for the solute-rich and poor phases does not change the free energy.

Interestingly, the equations that determine the binodal depend on the applied external pressure *p* and the overall, system-wide average concentration of solute in the system $\bar{\phi }$ (via $\bar{K}$ in the denominator). This behavior is markedly different from traditional phase-separating systems in the absence of an elastic medium (LLPS), where the concentrations of the coexisting phases depend only on the attractive forces between the particles and the temperature [31] and not the average concentration, due to the nature of the tie lines and lever rule. We discuss this feature and other unique properties of the binodal in the Discussion which follows.

The equations for the binodal also determine the mean field critical point of the phase diagram, which is where there is only one solution $\phi _\textrm{p}=\phi _\textrm{r}\equiv \phi _\textrm{c}$ of the two equations. The critical temperature (relative to the attraction) determines the minimal interaction strength between the solute molecules for which phase separation can occur. To find the critical point, we expand the coexisting concentrations $\phi _\textrm{p}$ and $\phi _\textrm{r}$ in Eqs. 7 and 8 for small deviations around the critical concentration $\phi _\textrm{c}$, which results in these two equations for the critical concentration and temperature (relative to the interaction strength, elastic constants and pressure).

9 $$\begin{aligned} f_\textrm{s}''\left( \phi _\textrm{c}\right)= & {} \frac{\left( \alpha ^{2}K^{2}-\beta ^{2}p^{2}\right) }{\left( K+\beta \phi _\textrm{c}\right) ^{2}\left( M+\beta \phi _\textrm{c}\right) }\end{aligned}$$

10 $$\begin{aligned} f_\textrm{s}'''\left( \phi _\textrm{c}\right)= & {} -\frac{3\beta \left( \alpha ^{2}K^{2}-\beta ^{2}p^{2}\right) }{\left( M+\beta \phi _\textrm{c}\right) ^{2}\left( K+\beta \phi _\textrm{c}\right) ^{2}} \end{aligned}$$

## Discussion

We have analyzed a system comprising gel containing a solute and solvent that can phase-separate within the gel. Both the single-phase and phase-separated states are encapsulated within the gel. The elastic properties of the gel which is coupled to the solute result in two important effects of the solute on the gel that are summarized in the equations for the binodal (Eqs. 7, 8). One is that the solute changes the hydration of the gel (e.g., leading to its expansion or contraction) via the parameter $\alpha$, and the second is that the solute changes the bulk modulus of the gel via the parameter $\beta$. When the solute phase separates, two concentric coexisting domains (an inner spherical core and an outer shell) form. The coupling of the solute concentration to the elasticity results in the fact that the gel in the core and shell can each contract or expand relative to the uniform, one-phase situation, which leads to inclusion-like behavior and induction of internal stresses and strains [33]. Furthermore, the bulk moduli of the core and shell increase or decrease relative to the one-phase system, which changes their elastic response to an external pressure *p*. Both these effects lead to changes in the elastic energy by phase separation, consequently impacting the equilibrium phase separation itself, as determined by the binodal. The extent of this impact depends on the reference mechanical properties of the one-phase state, which is determined by the overall solute concentration $\bar{\phi }$. The fact that the concentrations in the two coexisting states depend on the system-wide average composition, $\bar{\phi }$, is unique to the case in which the phase separation occurs in an elastic medium, which generates long-range effective interactions of the solute and solvent. While we considered a spherically symmetric sample, the phase separation can, in general, be more complex due to the long-range nature of elastic interactions, whose effects can depend on the geometry of the system, as demonstrated in the pioneering work of Wagner and Horner [4].

Equations 7 and 8 determine the binodal of a system with the simple and analytically solvable geometry of concentric coexisting domains and allow us to investigate the unique effects of *p* and $\bar{\phi }$ on the concentrations in each of the coexisting phases. We begin our analysis by first considering the case in which the solute does not change the bulk modulus of the gel ($\beta =0$) but still impacts the hydration and local expansion/contraction of the gel ($\alpha \ne 0$). Substituting $\beta =0$ into Eqs. 7 and 8 imposes that the bulk moduli of both phases are concentration-independent and hence equal. In this case, the binodal equations are:

11 $$\begin{aligned} \frac{f_\textrm{s}\left( \phi _\textrm{r}\right) -f_\textrm{s}\left( \phi _\textrm{p}\right) }{\phi _\textrm{r}-\phi _\textrm{p}}= & {} \frac{\partial f_\textrm{s}\left( \phi _\textrm{r}\right) }{\partial \phi _\textrm{r}}-\frac{1}{2}\frac{\alpha ^{2}}{M}\left( \phi _\textrm{r}-\phi _\textrm{p}\right) \end{aligned}$$

12 $$\begin{aligned} \frac{f_\textrm{s}\left( \phi _\textrm{r}\right) -f_\textrm{s}\left( \phi _\textrm{p}\right) }{\phi _\textrm{r}-\phi _\textrm{p}}= & {} \frac{\partial f_\textrm{s}\left( \phi _\textrm{p}\right) }{\partial \phi _\textrm{p}}+\frac{1}{2}\frac{\alpha ^{2}}{M}\left( \phi _\textrm{r}-\phi _\textrm{p}\right) \nonumber \\ \end{aligned}$$

We note that in the case of $\beta =0$, the second terms of right-hand side of the equations can be absorbed into the solute free energy $f_\textrm{s}$ by defining $\tilde{f}_\textrm{s}\left( \phi \right) =f_\textrm{s}\left( \phi \right) -\alpha ^{2}\phi ^{2}/\left( 2M\right)$. Therefore, if the bulk modulus is not affected by the solute, the expansion/contraction of the coexisting domains compared to the one-phase system effectively results in an additional two-body attraction of the solute molecules to each other that promotes phase separation (due to the negative sign). This attraction exists despite the fact that the elastic energy of the inclusion-like phase-separated domains is positive [33] because the energy of the local interaction between the solute and the gel polymer is reduced by the expansion and contraction of the phase-separated regions. The gel polymer thus mediates an effective attraction between the solutes independent of whether the gel polymers attract the solute (negative $\alpha$) and contract or repel the solute (positive $\alpha$) and swell, relative to their state in the absence of solute. In either case, the coupling of the elasticity and solute concentration means that phase separation is stabilized by the local contraction or expansion of the gel and vice versa. Interestingly, this implies that the presence of gel can possibly stabilize the phase separation of solutes that do not phase separate in the absence of the gel, highlighting the importance of the cellular environment (the cytoskeleton and the chromatin are both gels [22, 34]) for regulation of biomolecular phase separation.

Much richer behavior is found when the solute both interacts with the gel ($\alpha \ne 0$) and also modulates its mechanical properties ($\beta \ne 0$). As mentioned above, when $\beta \ne 0$, the external pressure *p* and the system-wide average solute concentration $\bar{\phi }$ determine the two coexisting concentrations given by the binodal equations. This results in two important properties.

**Fig. 2:**
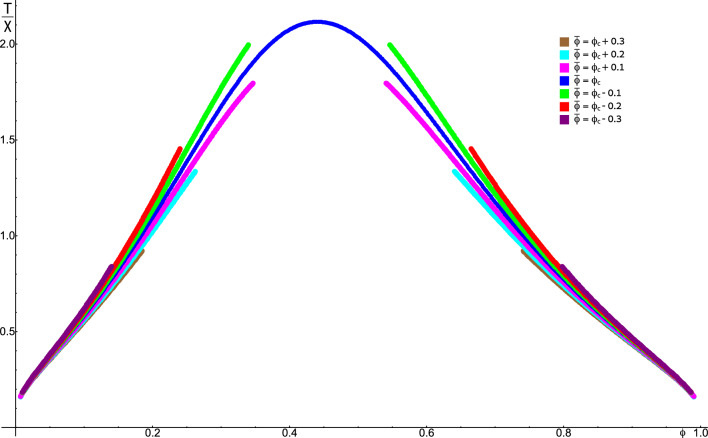
Binodal plots of the temperature (relative to the interaction strength) vs. the coexisting concentrations for different values of system-average solute concentration $\bar{\phi }$. Equations 7 and 8 were solved numerically for different values of $\bar{\phi }$ to demonstrate its effect on phase separation in gels, in contrast to fluid systems, where the coexisting concentrations are independent of $\bar{\phi }$. Seven values of $\bar{\phi }$ were chosen; the closed curve (dark blue) was the mean-field critical concentration predicted by Eqs. 9 and 10, along with three values of $\bar{\phi }$ below the critical concentration (the three outermost curves), and three values above it (innermost curves). For each value of $\bar{\phi }$, we plot the physical solutions of the equations for which $\bar{\phi }$ has a value between those of two coexisting concentrations since, otherwise, negative domain volumes are predicted by the relation between $\bar{\phi }$ and the coexisting concentrations. This condition leads to the discontinuous appearance of these curves (where $\bar{\phi }$ is not equal to the critical concentration of the system) since as the temperature increases, the difference between the two coexisting concentrations decreases. Thus, eventually, in all cases except when $\bar{\phi }$ equals the critical concentration, $\bar{\phi }$ becomes equal to one of the coexisting concentrations, implying that the volume of the other coexisting domain reaches zero. From the outer curves toward the inner ones, the values of $\bar{\phi }$ are $\phi _\textrm{c}-0.3$ (purple), $\phi _\textrm{c}-0.2$ (red), $\phi _\textrm{c}-0.1$ (green), $\phi _\textrm{c}$ (blue), $\phi _\textrm{c}+0.1$ (magenta), $\phi _\textrm{c}+0.2$ (cyan), and $\phi _\textrm{c}+0.3$ (Brown), where $\phi _\textrm{c}\approx 0.44$ is the mean-field critical concentration (including the elastic interactions). The contraction of the binodal with increasing $\bar{\phi }$ demonstrates its destabilizing effect on the phase-separated state. The parameters used for this plots are: bare bulk and shear moduli of $K=100$ kPa and $\mu =20$ kPa, respectively, which are representative of polyethylene glycol (PEG) [35], and $\alpha =\beta =25$ kPa were taken to be smaller but of a similar order of *K*; notably, for negative values of $\beta$, the binodal expands with increasing $\bar{\phi }$. The pressure was taken as $p=0$, which represents a stress-free boundary condition. The solution-free energy was taken as a lattice gas free energy [31], $f_\textrm{s}=k_\textrm{B}T\left( \phi \log \left( \phi \right) +\left( 1-\phi \right) \log \left( 1-\phi \right) \right) /v+\chi \phi \left( 1-\phi \right) /2v$, where $k_\textrm{B}T$ is the room temperature thermal energy, $\chi$ is the interaction strength, and $v=4000$ nm^3^ is the molecular volume taken to be that of a relatively large protein to better visualize the effects of elasticity on the phase separation

First, the coexisting solute concentrations depend on the average solute concentration, in contrast to the situation in the absence of the gel (LLPS). Specifically, as depicted in Fig. 2, for gels whose bulk modulus increases with the solute concentration ($\beta > 0$), an increase of the average solute concentration destabilizes the phase-separated state, shown by a reduction of the binodal area; if the solute decreases the bulk modulus ($\beta < 0$), the effect on the binodal is reversed. This can be explained by the elastic energy increase of an inclusion (which opposes phase separation) with the bulk modulus of the single-phase state. In the biological context, this means that phase separation of solutes that modulate the mechanical properties of gels around them does not necessarily “buffer” noise in the overall solute concentration (e.g., in an ensemble of cells or a single cell over a long time) [16]. Such buffering is one of the hypothesized physiological functions of LLPS and may not occur in the presence of a gel since the concentrations of the coexisting phases are not uniquely determined by the thermodynamics but, because of the long-range elastic interactions, depend on the average concentration of solute, $\bar{\phi }$, which varies in the ensemble or over long times. Interestingly, the long-range elastic interaction also renders the concept of tie lines irrelevant to the system of phase separation in a gel. Tie lines are defined as lines of equal chemical potential in the concentration–interaction phase space or alternatively as the locus of points in this phase space that give rise to phase-separated solutions with identical coexisting concentrations. In the usual fluid (non-gel) case, the coexisting concentrations are independent of a continuous range of value of the system-wide average concentration $\bar{\phi }$. In our case of phase separation in a gel, as shown in Fig. 2, variations of the system-wide average solute concentration $\bar{\phi }$ cause the binodal to shrink or expand so that a pair of coexisting concentrations uniquely determines the value of $\bar{\phi }$ (see SI section ”Invalidation of tie-lines by long-range elastic interactions” for mathematical proof). Therefore, only a single pair of $\bar{\phi }$ and interaction strength (temperature) lead to any given coexisting concentration. This conclusion implies that tie-lines cannot be generally defined in the presence of elastic long-range interactions coupled with phase separation; the values of the two coexisting concentrations depend on $\bar{\phi }$. This is in contrast to LLPS in the absence of a gel, where for each pair of coexisting concentrations away from the critical point, a continuous range of values of $\bar{\phi }$ give rise to these coexisting concentrations.

**Fig. 3:**
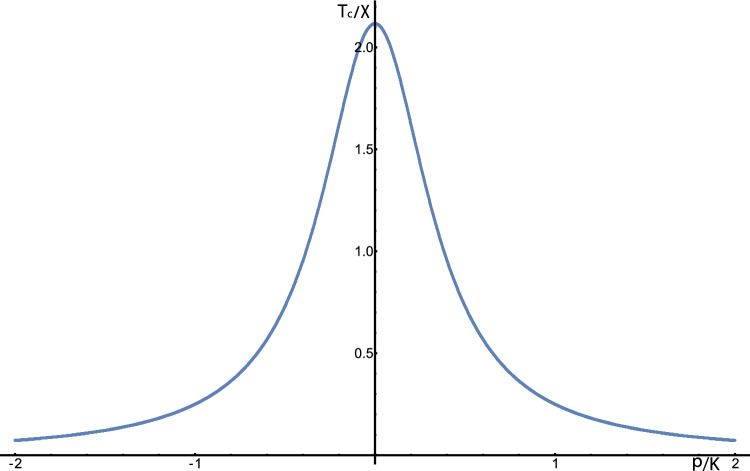
A plot of the critical temperature (relative to the interaction strength) vs. the external pressure (relative to the bare bulk modulus *K*). The plot is symmetric with respect to the inversion of the sign of the pressure, implying that mechanical compression and expansion have the same effect in our model, in which the free energy depends only on the magnitude of the pressure. The mean-field critical temperature (interaction strength) decreases (increases) with increasing magnitude of pressure, implying that the phase-separated state is destabilized. To solve Eqs. 9 and 10 to obtain the critical temperature, the parameters and solution-free energy used for Fig. 2 are employed, along with nonzero pressures varying from $-2K$ to $+2K$, where *K* is the bulk modulus in the absence of solute

Second, external mechanical forces can inhibit the phase separation of the liquid solution within the gel (see Fig. 3). This implies that phase-separated domains may sense stresses exerted on the surrounding gel. To understand this, we consider the mean-field critical point of the solvent–solute–gel. In the absence of the gel, the solute phase separation is driven by short-ranged, two-body attraction between pairs of solute molecules. The solution-free energy $f_\textrm{s}\left( \phi \right)$ can then be written as the sum of mixing entropy and the short-ranged, two-body interactions, $f_\textrm{s}\left( \phi \right) =S\left( \phi \right) -\chi \phi ^{2}/2$, where $S\left( \phi \right)$ is the mixing entropy of the solution and $\chi >0$ is the second virial coefficient of the solutes which represent the strength of the short-ranged attraction. We now add the effective interactions of the solutes induced by the elastic deformations of the gel. We substitute the expression for $f_\textrm{s}(\phi )$ into the equations for the critical point Eqs. 9 and 10 which results in explicit equations for the critical concentration $\phi _\textrm{c}$ and critical solute–solute interaction strength, $\chi _\textrm{c}$ (inversely proportional to the critical temperature in Fig. 3).

13 $$\begin{aligned}{} & {} S''\left( \phi _\textrm{c}\right) -\chi _\textrm{c} = \frac{\left( \alpha ^{2}K^{2}-\beta ^{2}p^{2}\right) }{\left( K+\beta \phi _\textrm{c}\right) ^{2}\left( M+\beta \phi _\textrm{c}\right) } \end{aligned}$$

14 $$\begin{aligned}{} & {} S'''\left( \phi _\textrm{c}\right) = -\frac{3\beta \left( \alpha ^{2}K^{2}-\beta ^{2}p^{2}\right) }{\left( M+\beta \phi _\textrm{c}\right) ^{2}\left( K+\beta \phi _\textrm{c}\right) ^{2}} \end{aligned}$$

Since $\beta$ represents the response of the bulk modulus to changes in the solute concentration, it is expected to be small compared to the modulus itself. Therefore, the right-hand side of Eq. 14, which determines the critical concentration, is small, and the critical concentration is expected to be close to the critical concentration in the absence of the gel and to be given by the equation $S'''\left( \phi _\textrm{c}\right) =0$. This is not necessarily true for the critical interaction strength $\chi _\textrm{c}$ which may be more sensitive to the coupling of the solute to the gel. In Eq. 13, which determines $\chi _\textrm{c}$, $\beta$ appears in the numerator of the right-hand side of the equation multiplied by the external pressure *p*, which can be large. Therefore, as given by Eq. 13, an increase of *p* inhibits phase separation since a *larger* value of the short-range interactions ($\chi _\textrm{c}>0$) will be required for the system to phase-separate. Furthermore, because *p* appears in the numerator as a squared quantity, only the absolute magnitude of the pressure rather than its sign impacts the critical interaction strength. The reason that modulation of the bulk moduli (represented by $\beta$) by the solute inhibits the phase separation is simple. Phase separation of the solute results in a structure consisting of an inner spherical core and a concentric shell, each of which have different bulk moduli. When such a structure is under pressure, its elastic energy increases with the difference of the bulk moduli of the two domains (see SI). Therefore, the contribution of modulation of the bulk modulus via the pressure increases the elastic energy of the phase-separated system and destabilizes the phase separation.

In contrast to the pressure *p*, and consistent with the discussion above, the interaction of the solute molecules with the polymer ($\alpha$) always allows phase separation to occur for *smaller* values of the short-range attraction, $\chi _\textrm{c}>0$ and thus stabilizes the phase separation. For small pressures ($\left| p\right| <\left| \alpha K/\beta \right|$), the stabilizing effects of the solute–polymer interaction dominate the inhibiting effects of the changes of the bulk modulus and the value of $\chi _\textrm{c}$ required for the system to phase-separate is smaller than in the absence of the gel. This trend reverses when $\left| p\right|$ is increased above $\left| \alpha K/\beta \right|$. However, when the two are equal, the stabilizing effect of the polymer–solute interaction and the destabilizing effect of the modulation of the bulk modulus, on the phase separation, cancel each other. Substitution of $\alpha K=\beta p$ in Eqs. 7 and 8 eliminates the second terms on the right-hand side of the equations, which results in the binodal of the solution in the absence of the gel.

The prediction of our model that pressure inhibits LLPS is consistent with experiments in in-vitro gels [8, 9]. However, the phase-separated domains in these experiments exclude the polymers. Our model predicts that the inhibitory effect of mechanical forces also remains valid when both phase-separated domains incorporate the gel polymer rather than exclude it. In biological, membrane-bound systems, the effect of mechanical forces may be more complex. In such cases, mechanical forces may inhibit phase separation of solutes that are in a gel (e.g., cytoskeleton, chromatin). However, these forces may also squeeze water from the region of the gel [36], leading to an increase in the concentration of the solute. In turn, this promotes phase separation, such as in the case of hyperosmotic phase separation [37]. The prospect of the non-monotonic effect of physical forces on LLPS in biological systems suggests a novel mechanism for mechanosensitivity. Many phase-separated biological condensates in the nucleus are involved in gene expression. These condensates, which are known as transcriptional condensates [17], may be sensitive to mechanical forces at the mesoscale via the effect of the forces on the formation or dissolution of the condensates, rather than the molecule itself being mechanosensitive [38]. The detailed interplay between mechanical forces exerted on a membrane-enclosed gel, dehydration of the gel, and the opposing effects of the forces on phase separation of solutes within the volume of the gel is outside of the scope of this work but is an interesting avenue for future research.

The unique nature of the solute we consider here that interacts with the gel polymer and changes its bulk modulus but not shear modulus is inspired by polyelectrolyte gels. In such gels, the local bulk modulus arises primarily from the osmotic pressure of counterions of the gel polymer. Consequently, the presence of charged solute changes the osmotic pressures and the bulk modulus of the gel [39]. These molecules can also bind the chromatin at a single site (monovalent in the language of LLPS), which gives rise to the chromatin–gel interactions ($\alpha$ in the free energy of 2), or at more than one site (multivalent), serving as cross-linkers that affect the shear modulus of the gel [27]. Here, we focused on these molecules that are not cross-linkers (i.e., monovalent), such as the high mobility group (HMG) proteins [40] or the protein BRD4 [41]. Naturally, in the case of cross-linking and phase-separating proteins (e.g., the nuclear heterochromatin protein 1 [42] or the cytoplasmic ARP2/3 complex [43]), the phase separation behavior is probably more complex than in the case we analyzed here analytically. Nonetheless, the prediction of our analytical, physical model presented here shows that including long-range mechanical interactions, which are prevalent in biological systems [44–46] in what is usually termed LLPS, but which may occur in a gel, results in qualitative properties that may be biologically meaningful. This includes the coupling between mechanical signals, the formation or inhibition of biological condensates, and the “violation” of concentration buffering [16]. The long-range elastic interactions that lead to these unique properties also characterize systems in which the phase-separating solute modulates the shear modulus (e.g., via cross-linkers that result in a local shape change). Thus, we expect qualitatively similar behavior of such systems, although they may differ quantitatively. The analysis of these systems is an interesting path for future research that is out of the scope of this paper. We believe this work will complement existing experimental studies, both in-vivo and in-vitro, focusing on phase separation within gels, where the phase-separated bodies exclude the gel polymer [8, 9, 11], and inspire future work on other systems, where the gel also permeates the phase-separated domains.

### Supplementary Information

Below is the link to the electronic supplementary material.

Supplementary file 1 (pdf 257 KB)

## Data Availability

All study data are included in the article and/or the electronic supplementary materials.
