# Supplementary material for: Mechanosensitivity of phase separation in an elastic gel
Source: Eur Phys J E Soft Matter. 2024 Feb 20;47(2):16. doi: 10.1140/epje/s10189-024-00405-y (PMC10879317; doi:10.1140/epje/s10189-024-00405-y)
Supplement: Supplementary file 1 — (pdf 257 KB) [file 10189_2024_405_MOESM1_ESM.pdf]

Supplementary Information: Mechanosensitivity of phase separation in an elastic gel

Dan Deviri[1,2] and Samuel A. Safran[1]

[1]Department of Chemical and Biological Physics, Weizmann Institute of Science, Rehovot 76100, Israel
[2]Carbon Blue Ltd., Haifa 3303201, Israel

Solution of the elastic problem in the presence of phase separated solution

We begin by writing the generalized stress tensor and force balance equation using a functional derivative of the phenomenological free energy F used in the main text [1]:

$$F = \int d^3\vec{r}\left(f_s(\phi) + \alpha\phi u_{\ell\ell} + \frac{\beta}{2}\phi u_{\ell\ell}^2 + \mu u_{ik}^2 + \frac{\lambda}{2}u_{\ell\ell}^2\right) \quad (1)$$

$$\sigma_{ij} = \frac{\delta F}{\delta u_{ij}} = \alpha\phi\delta_{ij} + (\lambda + \beta\phi)\, u_{\ell\ell}\delta_{ij} + 2\mu u_{ij} \quad (2)$$

$$\nabla\cdot\sigma = \partial_j\left(\alpha\phi\delta_{ij} + (\lambda+\beta\phi)\, u_{\ell\ell}\delta_{ij} + 2\mu u_{ij}\right) \quad (3)$$

$$= \nabla\left((\lambda+\mu+\beta\phi)(\nabla\cdot\vec{u}) + \alpha\phi\right) + \mu\nabla^2\vec{u} = 0 \quad (4)$$

where F is the free energy, ϕ is the volume fraction of the solute, $f_s(\phi)$ is the free energy density of the solution in the absence of the gel, and u_{ij} are the components of the strain tensor (where one sums over repeated indices). The quantities λ and μ are respectively, the renormalized first and second Lamé coefficients of the gel in the absence of solute, while α is a phenomenological constant related to the expansion or contraction of the surrounding elastic medium by the solute molecules, β is a phenomenological constant describing the change of bulk modulus of the gel due to the presence of the solute, and σ_{ij} are the components of the generalized stress tensor.

In a spherically symmetric case, the vector $\vec{u}$ depends only on the radial coordinate r so that

$$\nabla\cdot\vec{u} = \frac{du_r}{dr} + \frac{2u_r}{r} \quad (5)$$

$$\nabla^2\vec{u} = \frac{1}{r^2}\frac{\partial}{\partial r}\left(r^2\frac{\partial u_r}{\partial r}\right) - \frac{2u_r}{r^2} \quad (6)$$

where u_r is the radial component of the displacement vector and r is the radial coordinate. Substituting these two expressions into the force balance equation Eq. 3 results in an ordinary differential equation for the radial component of the displacement vector.

$$0 = (\lambda + 2\mu + \beta\phi)\left(\frac{d^2u_r}{dr^2} + \frac{2}{r}\frac{du_r}{dr} - \frac{2u_r}{r^2}\right) \Rightarrow \quad (7)$$

$$u_r = \frac{A}{3}r + \frac{B}{r^2} \quad (8)$$

where A and B are integration constants determined by the boundary conditions of the gel. We now write the non-vanishing components of the strain and stress tensors as functions of r and the integration constants A and B [2].

$$
\begin{aligned}
u_{rr} &= \frac{A}{3} - \frac{2B}{r^3} && (9)\\
u_{\theta\theta} &= u_{\phi\phi} = \frac{A}{3} + \frac{B}{r^3} \Rightarrow && (10)\\
\sigma_{rr} &= \alpha\phi + A\left(\lambda + \beta\phi\right) + 2\mu\left(\frac{A}{3} - \frac{2B}{r^3}\right) = \alpha\phi + A\left(K + \beta\phi\right) - \frac{4\mu B}{r^3} && (11)\\
\sigma_{\theta\theta} &= \sigma_{\phi\phi} = \alpha\phi + A\left(\lambda + \beta\phi\right) + 2\mu\left(\frac{A}{3} + \frac{B}{r^3}\right) = \alpha\phi + A\left(K + \beta\phi\right) + \frac{2\mu B}{r^3} && (12)
\end{aligned}
$$

where $K = \lambda + 2\mu/3$ is the bulk modulus of the gel. The strain and stresses above are valid for any spherically symmetric configuration of the system. We now focus on the phase separated configuration that is depicted in Fig. 1 in the main text and calculate the free energy of such configuration. As in the main text, we denote concentrations of the solute-rich (inner) and solute-poor (outer) phases by ϕ_r, ϕ_p respectively, the radius of the interface between the two phase by R_r, total size of the spherical gel by R_p. The equations for the four integration constants, A_r and B_r of the inner phase, and A_p and B_p of the outer phase, are:

1. Vanishing of the term proportional to $1/r^2$ in the displacement vector of the inner domain to avoid divergence of the displacement at $r = 0$:
$$B_r = 0 \qquad (13)$$

2. Equality of the radial stress, σ_{rr}, across the interface due to mechanical equilibrium, which is expressed as (see Eq. 2):
$$\alpha\phi_r + A_r\left(K + \beta\phi_r\right) = \alpha\phi_p + A_p\left(K + \beta\phi_p\right) - \frac{4\mu B_p}{R_r^3} \qquad (14)$$

3. Equality of the displacement vector across the interface, dictated by continuity of the displacement function. This is expressed as:
$$\frac{A_r}{3}R_r = \frac{A_p}{3}R_r + \frac{B_p}{R_r^2} \qquad (15)$$

4. The boundary condition at the outer surface of the gel. We consider two boundary conditions, a constant outer hydrostatic pressure p and zero displacement at $r = R_p$, which we now analyze in detail.

Zero displacement

For this boundary condition, the last equation for the integration constants is $u_r\left(r = R_p\right) = 0$, which results in the following expression:
$$\frac{A_p}{3}R_p + \frac{B_p}{R_p^2} = 0 \qquad (16)$$

The solution of the four equation for the integration constants in the case of zero displacement boundary condition, Eqs. 13, 14, 15, and 16 is:

$$
\begin{aligned}
A_r &= -\frac{\alpha\left(\phi_r - \phi_p\right)}{\left(M + \beta\left(\phi_r + \phi_p - \bar{\phi}\right)\right)}\frac{V_p}{V} && (17)\\
B_p &= -\frac{\alpha\left(\phi_r - \phi_p\right)}{3\left(M + \beta\left(\phi_r + \phi_p - \bar{\phi}\right)\right)}R_r^3 && (18)\\
A_p &= \frac{\alpha\left(\phi_r - \phi_p\right)}{\left(M + \beta\left(\phi_r + \phi_p - \bar{\phi}\right)\right)}\frac{V_r}{V} && (19)
\end{aligned}
$$

where $\bar{\phi}$ is the overall average concentration of the solute, $M = K + 4\mu/3$ is the p-wave modulus of the gel, $V_r = 4\pi R_r^3/3$ is the volume of the solute-rich phase, $V_p = 4\pi\left(R_p^3 - R_r^3\right)/3$ is the volume of the solute-poor phase, and $V = V_r + V_p$ is the total volume of the gel. Substituting these integration constants into the expressions for the displacement and strain components results in the following closed forms for the non-vanishing components of the displacement and strain:

$$u_r = \begin{cases} -\frac{\alpha(\phi_r-\phi_p)}{3\left(M+\beta\left(\phi_r+\phi_p-\bar{\phi}\right)\right)}\frac{V_p}{V}r & r \leq R_r \\ \frac{\alpha(\phi_r-\phi_p)}{3\left(M+\beta\left(\phi_r+\phi_p-\bar{\phi}\right)\right)}\frac{V_r}{V}r - \frac{\alpha(\phi_r-\phi_p)}{3\left(M+\beta\left(\phi_r+\phi_p-\bar{\phi}\right)\right)}\frac{R_r^3}{r^2} & R_r < r \leq R_p \end{cases} \tag{20}$$

$$u_{rr} = \begin{cases} -\frac{\alpha(\phi_r-\phi_p)}{3\left(M+\beta\left(\phi_r+\phi_p-\bar{\phi}\right)\right)}\frac{V_p}{V} & r \leq R_r \\ \frac{\alpha(\phi_r-\phi_p)}{3\left(M+\beta\left(\phi_r+\phi_p-\bar{\phi}\right)\right)}\frac{V_r}{V} + \frac{2\alpha(\phi_r-\phi_p)}{3\left(M+\beta\left(\phi_r+\phi_p-\bar{\phi}\right)\right)}\frac{R_r^3}{r^3} & R_r < r \leq R_p \end{cases} \tag{21}$$

$$u_{\theta\theta} = u_{\phi\phi} = \begin{cases} -\frac{\alpha(\phi_r-\phi_p)}{3\left(M+\beta\left(\phi_r+\phi_p-\bar{\phi}\right)\right)}\frac{V_p}{V} & r \leq R_r \\ \frac{\alpha(\phi_r-\phi_p)}{3\left(M+\beta\left(\phi_r+\phi_p-\bar{\phi}\right)\right)}\frac{V_r}{V} - \frac{\alpha(\phi_r-\phi_p)}{3\left(M+\beta\left(\phi_r+\phi_p-\bar{\phi}\right)\right)}\frac{R_r^3}{r^3} & R_r < r \leq R_p \end{cases} \Rightarrow \tag{22}$$

$$u_{\ell\ell} = u_{rr} + u_{\theta\theta} + u_{\phi\phi} = \begin{cases} -\frac{\alpha(\phi_r-\phi_p)}{\left(M+\beta\left(\phi_r+\phi_p-\bar{\phi}\right)\right)}\frac{V_p}{V} & r \leq R_r \\ \frac{\alpha(\phi_r-\phi_p)}{\left(M+\beta\left(\phi_r+\phi_p-\bar{\phi}\right)\right)}\frac{V_r}{V} & R_r < r \leq R_p \end{cases} \tag{23}$$

$$u_{ik}^2 = u_{rr}^2 + u_{\theta\theta}^2 + u_{\phi\phi}^2 = \begin{cases} \frac{1}{3}\left(\frac{\alpha(\phi_r-\phi_p)}{\left(M+\beta\left(\phi_r+\phi_p-\bar{\phi}\right)\right)}\frac{V_p}{V}\right)^2 & r < R_r \\ \frac{1}{3}\left(\frac{\alpha(\phi_r-\phi_p)}{\left(M+\beta\left(\phi_r+\phi_p-\bar{\phi}\right)\right)}\frac{V_r}{V}\right)^2 + \frac{6}{9}\left(\frac{\alpha(\phi_r-\phi_p)}{\left(M+\beta\left(\phi_r+\phi_p-\bar{\phi}\right)\right)}\frac{R_r^3}{r^3}\right)^2 & R_r < r < R_p \end{cases} \tag{24}$$

Next, we substitute the components of the strain tensor into the expression for the free energy in Eq. 1 and integrate, which results in the following expression for the total free energy:

$$\begin{aligned}
F &= \int d^3\vec{r}\left(f_s(\phi) + \alpha\phi u_{\ell\ell} + \frac{\beta}{2}\phi u_{\ell\ell}^2 + \mu u_{ik}^2 + \frac{\lambda}{2}u_{\ell\ell}^2\right) && (25)\\
&= V_r f_s(\phi_r) + V_p f_s(\phi_p) + \int_0^{R_r} 4\pi r^2 dr\left(\alpha\phi_r u_{\ell\ell} + \frac{\beta}{2}\phi_r u_{\ell\ell}^2 + \mu u_{ik}^2 + \frac{\lambda}{2}u_{\ell\ell}^2\right) && (26)\\
&+ \int_{R_r}^{R_p} 4\pi r^2 dr\left(\alpha\phi_p u_{\ell\ell} + \frac{\beta}{2}\phi_p u_{\ell\ell}^2 + \mu u_{ik}^2 + \frac{\lambda}{2}u_{\ell\ell}^2\right) && (27)\\
&= V_r f_s(\phi_r) - \alpha\phi_r\frac{\alpha(\phi_r-\phi_p)}{\left(M+\beta\left(\phi_r+\phi_p-\bar{\phi}\right)\right)}\frac{V_rV_p}{V} + \frac{1}{2}\frac{\alpha^2(K+\beta\phi_r)(\phi_r-\phi_p)^2}{\left(M+\beta\left(\phi_r+\phi_p-\bar{\phi}\right)\right)^2}\frac{V_rV_p^2}{V^2} && (28)\\
&+ V_p f_s(\phi_p) + \alpha\phi_p\left(\frac{\alpha(\phi_r-\phi_p)}{\left(M+\beta\left(\phi_r+\phi_p-\bar{\phi}\right)\right)}\right)\frac{V_rV_p}{V} + \frac{6\mu}{9}\left(\frac{\alpha(\phi_r-\phi_p)}{\left(M+\beta\left(\phi_r+\phi_p-\bar{\phi}\right)\right)}\right)^2\frac{V_rV_p}{V} && (29)\\
&+ \frac{1}{2}(K+\beta\phi_p)\left(\frac{\alpha(\phi_r-\phi_p)}{\left(M+\beta\left(\phi_r+\phi_p-\bar{\phi}\right)\right)}\right)^2\frac{V_r^2V_p}{V^2} && (30)\\
&= V_r f_s(\phi_r) + V_p f_s(\phi_p) - \frac{1}{2}\frac{\alpha^2(\phi_r-\phi_p)^2}{\left(M+\beta\left(\phi_r+\phi_p-\bar{\phi}\right)\right)}\frac{V_rV_p}{V} && (31)
\end{aligned}$$

We now minimize the total free energy above under the constraints of equal chemical potential and osmotic pressure which characterize coexisting phases [3] by introducing the grand potential $G = F - \eta\left(V_r\phi_r + V_p\phi_p\right) + \pi\left(V_r + V_p\right)$, where η and π are respectively the chemical potential and osmotic pressure. Minimization of G

leads to the equations that determine the binodal of the system (Eqs. 38 and 39 below):

$$G = V_r f_s(\phi_r) + V_p f_s(\phi_p) - \frac{\frac{1}{2}\alpha^2(\phi_r-\phi_p)^2}{M+\beta\left(\phi_r+\phi_p-\bar{\phi}\right)}\frac{V_r V_p}{V} - \eta\left(V_r\phi_r + V_p\phi_p\right) + \pi\left(V_r+V_p\right) \quad (32)$$

$$\frac{\partial G}{\partial \phi_r} = V_r\left(\frac{df_s(\phi_r)}{d\phi_r} - \frac{\alpha^2(\phi_r-\phi_p)}{\left(M+\beta\left(\phi_r+\phi_p-\bar{\phi}\right)\right)}\frac{V_p}{V} + \frac{1}{2}\frac{\beta\alpha^2(\phi_r-\phi_p)^2}{\left(M+\beta\left(\phi_r+\phi_p-\bar{\phi}\right)\right)^2}\frac{V_p}{V} - \eta\right) = 0 \quad (33)$$

$$\frac{\partial G}{\partial \phi_p} = V_p\left(\frac{df_s(\phi_p)}{d\phi_p} + \frac{\alpha^2(\phi_r-\phi_p)}{\left(M+\beta\left(\phi_r+\phi_p-\bar{\phi}\right)\right)}\frac{V_r}{V} + \frac{1}{2}\frac{\beta\alpha^2(\phi_r-\phi_p)^2}{\left(M+\beta\left(\phi_r+\phi_p-\bar{\phi}\right)\right)^2}\frac{V_r}{V} - \eta\right) = 0 \quad (34)$$

$$\frac{\partial G}{\partial V_r} = f_s(\phi_r) - \frac{1}{2}\frac{\alpha^2(\phi_r-\phi_p)^2}{\left(M+\beta\left(\phi_r+\phi_p-\bar{\phi}\right)\right)}\frac{V_p}{V} - \eta\phi_r + \pi = 0 \quad (35)$$

$$\frac{\partial G}{\partial V_p} = f_s(\phi_p) - \frac{1}{2}\frac{\alpha^2(\phi_r-\phi_p)^2}{\left(M+\beta\left(\phi_r+\phi_p-\bar{\phi}\right)\right)}\frac{V_r}{V} - \eta\phi_p + \pi = 0 \Rightarrow \quad (36)$$

$$\eta = \frac{f_s(\phi_r)-f_s(\phi_p)}{\phi_r-\phi_p} - \frac{1}{2}\frac{\alpha^2(\phi_r-\phi_p)}{\left(M+\beta\left(\phi_r+\phi_p-\bar{\phi}\right)\right)}\frac{V_p-V_r}{V} \Rightarrow \quad (37)$$

$$\frac{f_s(\phi_r)-f_s(\phi_p)}{\phi_r-\phi_p} = \frac{df_s(\phi_r)}{d\phi_r} - \frac{1}{2}\frac{\alpha^2(M+\beta\phi_p)(\phi_r-\phi_p)}{\left(M+\beta\left(\phi_r+\phi_p-\bar{\phi}\right)\right)^2} \quad (38)$$

$$\frac{f_s(\phi_r)-f_s(\phi_p)}{\phi_r-\phi_p} = \frac{df_s(\phi_p)}{d\phi_p} + \frac{1}{2}\frac{\alpha^2(M+\beta\phi_r)(\phi_r-\phi_p)}{\left(M+\beta\left(\phi_r+\phi_p-\bar{\phi}\right)\right)^2} \quad (39)$$

Constant outer hydrostatic pressure

For this boundary condition, the last condition for the integration constants is $\sigma_{rr}(r = R_p) = p$, which results in the following expression:

$$\alpha\phi_p + A_p(K+\beta\phi_p) - \frac{4\mu B_p}{R_p^3} = p \quad (40)$$

The solution of the four equations for the integration constants in the case of constant outer hydrostatic pressure boundary condition, Eqs. 13, 14, 15, and 40 is:

$$A_p = \frac{(K+\beta\phi_r)(p-\alpha\phi_p) + \frac{4\mu}{3}\left(p-\alpha\bar{\phi}\right)}{(K+\beta\phi_r)(K+\beta\phi_p) + \frac{4\mu}{3}\left(K+\beta\bar{\phi}\right)} \quad (41)$$

$$B_p = -\frac{(K\alpha+\beta p)(\phi_r-\phi_p)}{(K+\beta\phi_r)(K+\beta\phi_p) + \frac{4\mu}{3}\left(K+\beta\bar{\phi}\right)}\frac{R_r^3}{3} \quad (42)$$

$$A_r = \frac{(K+\beta\phi_p)(p-\alpha\phi_r) + \frac{4\mu}{3}\left(p-\alpha\bar{\phi}\right)}{(K+\beta\phi_r)(K+\beta\phi_p) + \frac{4\mu}{3}\left(K+\beta\bar{\phi}\right)} \quad (43)$$

where $\bar{\phi}$ is the overall average concentration of the solute. Substituting these integration constants into the expressions for the displacement in Eq. 8, and using this to calculate the components of the strain tensor [2] results in the following expressions:

$$u_r = \begin{cases} \frac{1}{3}\frac{(K+\beta\phi_p)(p-\alpha\phi_r)+\frac{4\mu}{3}\left(p-\alpha\bar{\phi}\right)}{(K+\beta\phi_r)(K+\beta\phi_p)+\frac{4\mu}{3}\left(K+\beta\bar{\phi}\right)}r & r < R_r \\ \frac{r}{3}\left(\frac{(K+\beta\phi_r)(p-\alpha\phi_p)+\frac{4\mu}{3}\left(p-\alpha\bar{\phi}\right)-(\alpha K+\beta p)(\phi_r-\phi_p)\frac{R_r^3}{r^3}}{(K+\beta\phi_r)(K+\beta\phi_p)+\frac{4\mu}{3}\left(K+\beta\bar{\phi}\right)}\right) & R_r < r < R_p \end{cases} \quad (44)$$

$$u_{rr} = \begin{cases} \frac{1}{3}\frac{(K+\beta\phi_p)(p-\alpha\phi_r)+\frac{4\mu}{3}\left(p-\alpha\bar{\phi}\right)}{(K+\beta\phi_r)(K+\beta\phi_p)+\frac{4\mu}{3}\left(K+\beta\bar{\phi}\right)} & r<R_r \\ \frac{1}{3}\left(\frac{(K+\beta\phi_r)(p-\alpha\phi_p)+\frac{4\mu}{3}\left(p-\alpha\bar{\phi}\right)+2(\alpha K+\beta p)(\phi_r-\phi_p)\frac{R_r^3}{r^3}}{(K+\beta\phi_r)(K+\beta\phi_p)+\frac{4\mu}{3}\left(K+\beta\bar{\phi}\right)}\right) & R_r<r<R_p \end{cases} \tag{45}$$

$$u_{\theta\theta} = u_{\phi\phi} = \begin{cases} \frac{1}{3}\frac{(K+\beta\phi_p)(p-\alpha\phi_r)+\frac{4\mu}{3}\left(p-\alpha\bar{\phi}\right)}{(K+\beta\phi_r)(K+\beta\phi_p)+\frac{4\mu}{3}\left(K+\beta\bar{\phi}\right)} & r<R_r \\ \frac{1}{3}\left(\frac{(K+\beta\phi_r)(p-\alpha\phi_p)+\frac{4\mu}{3}\left(p-\alpha\bar{\phi}\right)-(\alpha K+\beta p)(\phi_r-\phi_p)\frac{R_r^3}{r^3}}{(K+\beta\phi_r)(K+\beta\phi_p)+\frac{4\mu}{3}\left(K+\beta\bar{\phi}\right)}\right) & R_r<r<R_p \end{cases} \Rightarrow \tag{46}$$

$$u_{\ell\ell} = \begin{cases} \frac{(K+\beta\phi_p)(p-\alpha\phi_r)+\frac{4\mu}{3}\left(p-\alpha\bar{\phi}\right)}{(K+\beta\phi_r)(K+\beta\phi_p)+\frac{4\mu}{3}\left(K+\beta\bar{\phi}\right)} & r<R_r \\ \frac{(K+\beta\phi_r)(p-\alpha\phi_p)+\frac{4\mu}{3}\left(p-\alpha\bar{\phi}\right)}{(K+\beta\phi_r)(K+\beta\phi_p)+\frac{4\mu}{3}\left(K+\beta\bar{\phi}\right)} & R_r<r<R_p \end{cases} \tag{47}$$

$$u_{ik}^2 = \begin{cases} \frac{1}{3}\left(\frac{(K+\beta\phi_p)(p-\alpha\phi_r)+\frac{4\mu}{3}\left(p-\alpha\bar{\phi}\right)}{(K+\beta\phi_r)(K+\beta\phi_p)+\frac{4\mu}{3}\left(K+\beta\bar{\phi}\right)}\right)^2 & r<R_r \\ \frac{1}{3}\left(\frac{(K+\beta\phi_r)(p-\alpha\phi_p)+\frac{4\mu}{3}\left(p-\alpha\bar{\phi}\right)}{(K+\beta\phi_r)(K+\beta\phi_p)+\frac{4\mu}{3}\left(K+\beta\bar{\phi}\right)}\right)^2+\frac{2}{3}\left(\frac{(\alpha K+\beta p)(\phi_r-\phi_p)\frac{R_r^3}{r^3}}{(K+\beta\phi_r)(K+\beta\phi_p)+\frac{4\mu}{3}\left(K+\beta\bar{\phi}\right)}\right)^2 & R_r<r<R_p \end{cases} \tag{48}$$

Next, we substitute the components of the strain tensor into Eq. 1 to express the free energy density as functions of the elastic constants, solute concentrations in each phase, the overall average solute concentration and the pressure:

$$f \tag{49}$$

$$= f_s(\phi)+\alpha\phi u_{\ell\ell}+\frac{\beta}{2}\phi u_{\ell\ell}^2+\mu u_{ik}^2+\frac{\lambda}{2}u_{\ell\ell}^2 \tag{50}$$

$$= \begin{cases} f_s(\phi_r)+\alpha\phi_r\left(\frac{(K+\beta\phi_p)(p-\alpha\phi_r)+\frac{4\mu}{3}\left(p-\alpha\bar{\phi}\right)}{(K+\beta\phi_r)(K+\beta\phi_p)+\frac{4\mu}{3}\left(K+\beta\bar{\phi}\right)}\right)+\frac{1}{2}(K+\beta\phi_r)\frac{\left((K+\beta\phi_p)(p-\alpha\phi_r)+\frac{4\mu}{3}\left(p-\alpha\bar{\phi}\right)\right)^2}{\left((K+\beta\phi_r)(K+\beta\phi_p)+\frac{4\mu}{3}\left(K+\beta\bar{\phi}\right)\right)^2} & r<R_r \\ f_s(\phi_p)+\alpha\phi_p\left(\frac{(K+\beta\phi_r)(p-\alpha\phi_p)+\frac{4\mu}{3}\left(p-\alpha\bar{\phi}\right)}{(K+\beta\phi_r)(K+\beta\phi_p)+\frac{4\mu}{3}\left(K+\beta\bar{\phi}\right)}\right)+\frac{1}{2}(K+\beta\phi_p)\frac{\left((K+\beta\phi_r)(p-\alpha\phi_p)+\frac{4\mu}{3}\left(p-\alpha\bar{\phi}\right)\right)^2}{\left((K+\beta\phi_r)(K+\beta\phi_p)+\frac{4\mu}{3}\left(K+\beta\bar{\phi}\right)\right)^2} & \\ \qquad +\frac{2\mu}{3}\left(\frac{(\alpha K+\beta p)(\phi_r-\phi_p)\frac{R_r^3}{r^3}}{(K+\beta\phi_r)(K+\beta\phi_p)+\frac{4\mu}{3}\left(K+\beta\bar{\phi}\right)}\right)^2 & R_r<r<R_p \end{cases} \tag{51}$$

Integrating this free energy density over the volume of the gel results in the total free energy of the system:

$$F = V_r f_s(\phi_r)+\alpha\phi_r\left(\frac{(K+\beta\phi_p)(p-\alpha\phi_r)+\frac{4\mu}{3}\left(p-\alpha\bar{\phi}\right)}{(K+\beta\phi_r)(K+\beta\phi_p)+\frac{4\mu}{3}\left(K+\beta\bar{\phi}\right)}\right)V_r \tag{52}$$

$$+ \frac{1}{2}(K+\beta\phi_r)\left(\frac{(K+\beta\phi_p)(p-\alpha\phi_r)+\frac{4\mu}{3}\left(p-\alpha\bar{\phi}\right)}{(K+\beta\phi_r)(K+\beta\phi_p)+\frac{4\mu}{3}\left(K+\beta\bar{\phi}\right)}\right)^2 V_r \tag{53}$$

$$+ V_p f_s(\phi_p)+\alpha\phi_p\left(\frac{(K+\beta\phi_r)(p-\alpha\phi_p)+\frac{4\mu}{3}\left(p-\alpha\bar{\phi}\right)}{(K+\beta\phi_r)(K+\beta\phi_p)+\frac{4\mu}{3}\left(K+\beta\bar{\phi}\right)}\right)V_p \tag{54}$$

$$+ +\frac{1}{2}(K+\beta\phi_p)\left(\frac{(K+\beta\phi_r)(p-\alpha\phi_p)+\frac{4\mu}{3}\left(p-\alpha\bar{\phi}\right)}{(K+\beta\phi_r)(K+\beta\phi_p)+\frac{4\mu}{3}\left(K+\beta\bar{\phi}\right)}\right)^2 V_p \tag{55}$$

$$+ \frac{2\mu}{3}\left(\frac{(\alpha K+\beta p)(\phi_r-\phi_p)}{(K+\beta\phi_r)(K+\beta\phi_p)+\frac{4\mu}{3}\left(K+\beta\bar{\phi}\right)}\right)^2\frac{V_r V_p}{V} \tag{56}$$

Similar to the calculation in the subsection "Zero displacement", we write the grand potential $G = F - \eta(V_r\phi_r + V_p\phi_p) + \pi(V_r + V_p)$ and minimize it with respect to the coexisting concentrations ϕ_r and ϕ_p, and

coexisting volumes V_r and V_p, to determine the binodal of the system [3].

$$G = V_r\left(\frac{\alpha\phi_r\left(\left(K+\beta\phi_p\right)\left(p-\alpha\phi_r\right)+\frac{4\mu}{3}\left(p-\alpha\bar{\phi}\right)\right)}{\left(\left(K+\beta\phi_r\right)\left(K+\beta\phi_p\right)+\frac{4\mu}{3}\left(K+\beta\bar{\phi}\right)\right)}+\frac{\left(K+\beta\phi_r\right)\left(\left(K+\beta\phi_p\right)\left(p-\alpha\phi_r\right)+\frac{4\mu}{3}\left(p-\alpha\bar{\phi}\right)\right)^2}{2\left(\left(K+\beta\phi_r\right)\left(K+\beta\phi_p\right)+\frac{4\mu}{3}\left(K+\beta\bar{\phi}\right)\right)^2}\right) \quad (57)$$

$$+ V_p\left(\frac{\alpha\phi_p\left(\left(K+\beta\phi_r\right)\left(p-\alpha\phi_p\right)+\frac{4\mu}{3}\left(p-\alpha\bar{\phi}\right)\right)}{\left(\left(K+\beta\phi_r\right)\left(K+\beta\phi_p\right)+\frac{4\mu}{3}\left(K+\beta\bar{\phi}\right)\right)}+\frac{\left(K+\beta\phi_p\right)\left(\left(K+\beta\phi_r\right)\left(p-\alpha\phi_p\right)+\frac{4\mu}{3}\left(p-\alpha\bar{\phi}\right)\right)^2}{2\left(\left(K+\beta\phi_r\right)\left(K+\beta\phi_p\right)+\frac{4\mu}{3}\left(K+\beta\bar{\phi}\right)\right)^2}\right) \quad (58)$$

$$+ V_r f_s\left(\phi_r\right)+V_p f_s\left(\phi_p\right)+\frac{\frac{2\mu}{3}\alpha^2\left(K+\frac{\beta p}{\alpha}\right)^2\left(\phi_r-\phi_p\right)^2\frac{V_rV_p}{V}}{\left(\left(K+\beta\phi_r\right)\left(K+\beta\phi_p\right)+\frac{4\mu}{3}\left(K+\beta\bar{\phi}\right)\right)^2}-\eta\left(V_r\phi_r+V_p\phi_p\right)+\pi\left(V_r+V_p\right) \quad (59)$$

$$\frac{\partial G}{\partial V_r} = \alpha\phi_r\left(\frac{\left(K+\beta\phi_p\right)\left(p-\alpha\phi_r\right)+\frac{4\mu}{3}\left(p-\alpha\bar{\phi}\right)}{\left(K+\beta\phi_r\right)\left(K+\beta\phi_p\right)+\frac{4\mu}{3}\left(K+\beta\bar{\phi}\right)}\right)+\frac{1}{2}\left(K+\beta\phi_r\right)\left(\frac{\left(K+\beta\phi_p\right)\left(p-\alpha\phi_r\right)+\frac{4\mu}{3}\left(p-\alpha\bar{\phi}\right)}{\left(K+\beta\phi_r\right)\left(K+\beta\phi_p\right)+\frac{4\mu}{3}\left(K+\beta\bar{\phi}\right)}\right)^2 \quad (60)$$

$$+ \frac{2\mu}{3}\left(\frac{\alpha\left(K+\frac{\beta p}{\alpha}\right)\left(\phi_r-\phi_p\right)}{\left(K+\beta\phi_r\right)\left(K+\beta\phi_p\right)+\frac{4\mu}{3}\left(K+\beta\bar{\phi}\right)}\right)^2\frac{V_p}{V}-\eta\phi_r+\pi+f_s\left(\phi_r\right)=0 \quad (61)$$

$$\frac{\partial G}{\partial V_p} = \alpha\phi_p\left(\frac{\left(K+\beta\phi_r\right)\left(p-\alpha\phi_p\right)+\frac{4\mu}{3}\left(p-\alpha\bar{\phi}\right)}{\left(K+\beta\phi_r\right)\left(K+\beta\phi_p\right)+\frac{4\mu}{3}\left(K+\beta\bar{\phi}\right)}\right)+\frac{1}{2}\left(K+\beta\phi_p\right)\left(\frac{\left(K+\beta\phi_r\right)\left(p-\alpha\phi_p\right)+\frac{4\mu}{3}\left(p-\alpha\bar{\phi}\right)}{\left(K+\beta\phi_r\right)\left(K+\beta\phi_p\right)+\frac{4\mu}{3}\left(K+\beta\bar{\phi}\right)}\right)^2 \quad (62)$$

$$+ \frac{2\mu}{3}\left(\frac{\alpha\left(K+\frac{\beta p}{\alpha}\right)\left(\phi_r-\phi_p\right)}{\left(K+\beta\phi_r\right)\left(K+\beta\phi_p\right)+\frac{4\mu}{3}\left(K+\beta\bar{\phi}\right)}\right)^2\frac{V_r}{V}-\eta\phi_p+\pi+f_s\left(\phi_p\right)=0\Rightarrow \quad (63)$$

$$\eta = \frac{f_s\left(\phi_r\right)-f_s\left(\phi_p\right)}{\phi_r-\phi_p}+\left(\frac{\alpha\left(K\left(p-\alpha\left(\phi_r+\phi_p\right)\right)-\alpha\beta\phi_r\phi_p\right)+\frac{4\mu}{3}\alpha\left(p-\alpha\bar{\phi}\right)}{\left(K+\beta\phi_r\right)\left(K+\beta\phi_p\right)+\frac{4\mu}{3}\left(K+\beta\bar{\phi}\right)}\right) \quad (64)$$

$$- \frac{\frac{4\mu}{3}\alpha\left(K+\beta\phi_r\right)\left(K+\beta\phi_p\right)\left(p-\alpha\bar{\phi}\right)}{\left(\left(K+\beta\phi_r\right)\left(K+\beta\phi_p\right)+\frac{4\mu}{3}\left(K+\beta\bar{\phi}\right)\right)^2} \quad (65)$$

$$+ \frac{1}{2}\left(\frac{\left(K+\beta\phi_r\right)\left(K+\beta\phi_p\right)\left(-\beta p^2-2p\alpha K+\alpha^2\left(K\left(\phi_r+\phi_p\right)+\beta\phi_r\phi_p\right)\right)}{\left(\left(K+\beta\phi_r\right)\left(K+\beta\phi_p\right)+\frac{4\mu}{3}\left(K+\beta\bar{\phi}\right)\right)^2}\right) \quad (66)$$

$$+ \frac{\frac{\beta}{2}\left(\frac{4\mu}{3}\left(p-\alpha\bar{\phi}\right)\right)^2}{\left(\left(K+\beta\phi_r\right)\left(K+\beta\phi_p\right)+\frac{4\mu}{3}\left(K+\beta\bar{\phi}\right)\right)^2} \quad (67)$$

$$+ \frac{2\mu}{3}\frac{\left(K\alpha+\beta p\right)^2\left(\phi_r-\phi_p\right)}{\left(\left(K+\beta\phi_r\right)\left(K+\beta\phi_p\right)+\frac{4\mu}{3}\left(K+\beta\bar{\phi}\right)\right)^2}\left(\frac{V_p}{V}-\frac{V_r}{V}\right) \quad (68)$$

$$\frac{\partial G}{\partial \phi_r} = 0\Rightarrow \quad (69)$$

$$\frac{f_s\left(\phi_r\right)-f_s\left(\phi_p\right)}{\phi_r-\phi_p}=\frac{\partial f_s\left(\phi_r\right)}{\partial\phi_r}-\frac{1}{2}\frac{\left(K^2\alpha^2-p^2\beta^2\right)\left(M+\beta\phi_p\right)\left(\phi_r-\phi_p\right)}{\left(\left(K+\beta\phi_r\right)\left(K+\beta\phi_p\right)+\frac{4\mu}{3}\left(K+\beta\bar{\phi}\right)\right)^2} \quad (70)$$

$$\frac{\partial G}{\partial \phi_p} = 0\Rightarrow \quad (71)$$

$$\frac{f_s\left(\phi_r\right)-f_s\left(\phi_p\right)}{\phi_r-\phi_p}=\frac{\partial f_s\left(\phi_p\right)}{\partial\phi_p}+\frac{1}{2}\frac{\left(K^2\alpha^2-p^2\beta^2\right)\left(M+\beta\phi_r\right)\left(\phi_r-\phi_p\right)}{\left(\left(K+\beta\phi_r\right)\left(K+\beta\phi_p\right)+\frac{4\mu}{3}\left(K+\beta\bar{\phi}\right)\right)^2} \quad (72)$$

To conclude, the binodal equations of the system are:

$$\frac{f_s(\phi_r)-f_s(\phi_p)}{\phi_r-\phi_p} = \frac{\partial f_s(\phi_r)}{\partial \phi_r}-\frac{1}{2}\frac{\left(K^2\alpha^2-p^2\beta^2\right)(M+\beta\phi_p)(\phi_r-\phi_p)}{\left((K+\beta\phi_r)(K+\beta\phi_p)+\frac{4\mu}{3}\left(K+\beta\bar{\phi}\right)\right)^2} \tag{73}$$

$$\frac{f_s(\phi_r)-f_s(\phi_p)}{\phi_r-\phi_p} = \frac{\partial f_s(\phi_p)}{\partial \phi_p}+\frac{1}{2}\frac{\left(K^2\alpha^2-p^2\beta^2\right)(M+\beta\phi_r)(\phi_r-\phi_p)}{\left((K+\beta\phi_r)(K+\beta\phi_p)+\frac{4\mu}{3}\left(K+\beta\bar{\phi}\right)\right)^2} \tag{74}$$

Solution for elastic problem of concentric core and shell under hydrostatic pressure p

In this subsection, we express the energy of a concentric elastic core and shell which have different bulk moduli but the same shear moduli, and which are both subject to hydrostatic pressure p. The different elastic moduli are the result of the phase separation of solute in the core and shell since the solute concentration modulates the bulk modulus of the gel. This model generates insights that contribute to the understanding of the results of the more complex phase separation model in the main text.

Consistent with the notation used in the main text (see Fig. 1 in the main text), we denote the bulk moduli of the inner core and the outer shell as K_r and K_p, respectively. Similarly, the radius of the inner core is denoted by R_r and of the outer shell (and the entire spherical gel), by R_p. We now write the equations that determine the deformation of the gel. Since there are no body forces acting on the gel, the displacement vector $\vec{u}$ satisfies the following equation [2]:

$$(1-2\nu)\,\Delta\vec{u}+\nabla(\nabla\cdot\vec{u})=0 \tag{75}$$

where $\nu=\left(K-\frac{2\mu}{3}\right)/\left(2K+\frac{2\mu}{3}\right)$ is the Poisson ratio; K and μ are respectively the bulk and shear moduli. In a spherically symmetric system such as the one we discuss, the displacement vector must have only radial component that depends only on the radial coordinate, $\vec{u}=u_r(r)\,\hat{r}$. Substituting this form of the displacement vector into Eq. 75 results in the same ordinary differential equation and solution as in the previous subsection:

$$0 = 2(1-\nu)\left(\frac{d^2u_r}{dr^2}+\frac{2}{r}\frac{du_r}{dr}-\frac{2u_r}{r^2}\right)\Rightarrow \tag{76}$$

$$u_r = Ar+\frac{B}{r^2} \tag{77}$$

where A and B are integration constants that are determined by boundary conditions. Since the core and the shell have different bulk moduli, the integration constants can differ; we therefore denote by A_r and B_r the constant for the core, and by A_p and B_p the constants for the shell. As in the previous subsection, the the four integration constants are determined by the following conditions:

1. Vanishing of the term proportional to $1/r^2$ in the expression for the displacement vector of the inner domain to avoid divergence of the displacement at $r=0$:

$$B_r=0 \tag{78}$$

2. Equality of σ_{rr} across the interface due to mechanical equilibrium (force balance) [2]:

$$3K_rA_r=3K_pA_p-4\mu\frac{B_p}{R_r^3} \tag{79}$$

3. Equality of the displacement vector across the interface, dictated by continuity of the displacement function:

$$A_rR_r=A_pR_r+\frac{B_p}{R_r^2} \tag{80}$$

4. A constant hydrostatic pressure p that acts upon the outer surface of the spherical shell (e.g., by a fluid that is immiscible with the gel and the solution contained within it):

$$3K_pA_p - 4\mu\frac{B_p}{R_p^3} = p \tag{81}$$

The solution of these three equations results in the four integration constants:

$$A_r = \frac{M_p p}{3\left(K_pK_r + \frac{4\mu}{3}\left(K_p\frac{V_p}{V} + K_r\frac{V_r}{V}\right)\right)} \tag{82}$$

$$A_p = \frac{M_r p}{3\left(K_pK_r + \frac{4\mu}{3}\left(K_p\frac{V_p}{V} + K_r\frac{V_r}{V}\right)\right)} \tag{83}$$

$$B_p = \frac{(K_p - K_r)\,p}{3\left(K_pK_r + \frac{4\mu}{3}\left(K_p\frac{V_p}{V} + K_r\frac{V_r}{V}\right)\right)}R_r^3 \tag{84}$$

where as before, V_r and V_p, are the respective volumes of the core and the shell, $V = V_r + V_p$ is the total volume of the spherical elastic material, and $M_i = K_i + 4\mu/3$ is the p-wave modulus of the core or shell ($i = 1, 2$, respectively). To find the elastic energy E of the core and shell under pressure p, we first substitute these integration constant into the displacement (Eq. 77). Then, we calculate the components of the strain tensor and substitute these components into the expression for the energy density of a linear elastic system and integrate [2]:

$$E = \int d^3\vec{r}\left(\mu u_{ik}^2 + \frac{\lambda}{2}u_{\ell\ell}^2\right) = \int_0^{R_r} d^3\vec{r}\left(\frac{9}{2}K_rA_r^2\right) + \int_{R_r}^{R_p} d^3\vec{r}\left(\frac{9}{2}K_pA_p^2 + 6\mu\frac{B_p^2}{r^6}\right) \tag{85}$$

$$= \frac{1}{2}\frac{p^2\left(\left(M_p^2K_rV_r + M_r^2K_pV_p\right) + \frac{4\mu}{3}\left(K_p - K_r\right)^2\frac{V_rV_p}{V}\right)}{\left(K_pK_r + \frac{4\mu}{3}\bar{K}\right)^2} \tag{86}$$

where $\bar{K} = \left(K_rV_r + K_pV_p\right)/V$ is the volumetric average of the bulk moduli. For the case considered in the main text, the volumetric average of the bulk moduli is a constant determined by the (overall average) concentration of solute $\bar{\phi}$. Under the constraint of fixed $\bar{K}$, we can now investigate the effect of increasing the difference between the bulk moduli of the core and the shell due to the effect of the solute on the gel mechanics, by substituting into the expression for the elastic energy in Eq. 86 the expansions $K_r = \bar{K} + V\delta/V_r$ and $K_p = \bar{K} - V\delta/V_p$. Here, δ is a parameter related to β and the concentration of the solute in the two compartment that represents the magnitude of the change in the bulk moduli compared to the one-phase state of the system; importantly, this expansion conserves the relation $\bar{K} = \left(K_rV_r + K_pV_p\right)/V$.

Expansion of the elastic energy of Eq. 86 with respect to the parameter δ results in:

$$E = \frac{p^2}{2\bar{K}}V + \frac{p^2V^3}{2\bar{K}^2V_pV_r\left(\bar{K} + \frac{4\mu}{3}\right)}\delta^2 + \frac{p^2\left(V_r - V_p\right)V^4}{2\bar{K}^2V_p^2V_r^2\left(\bar{K} + \frac{4\mu}{3}\right)^2}\delta^3 + O\left(\delta^4\right) \tag{87}$$

The vanishing of the first order term in δ and the fact that the second order term in δ is positive, suggests that for modulation of the bulk modulus by the solute that is small compared to the bulk modulus itself, the term in δ^2 that is induced by phase separation increases the elastic energy by an amount proportional to p^2. This implies that the external pressure destabilizes phase separation, as predicted by our more detailed model that is presented in the main text.

Invalidation of tie-lines by long-range elastic interactions

In phase-separating systems in the absence of long-range elastic interactions, it is convenient to define tie-lines as lines in the interaction-concentration phase space describing a constant chemical potential of the system [3].

An equivalent and helpful definition of the tie-lines is the locus of points in the phase space of interaction and average concentration (or concentrations in multi-component systems), which account for phase separation into domains of similar coexisting concentrations but potentially different volumes. In the fluid case (with no gel), the coexisting concentrations are independent of the system-wide average concentration, $\bar{\phi}$; only the relative volumes of the two phases depend on $\bar{\phi}$. Such a graphical descriptionis commonly used in the field of biological phase separation [4]. This section investigates the form of the tie-lines for phase separation of a solution in a gel coupled to long-range elastic interactions of the gel. As we conclude below, these long-range interactions result in a one–one dependence between the coexisting concentration and the *average, system-wide concentration* and solute-solute interaction energy, thereby invalidating the notion of the tie-lines. To investigate this further, we substitute into Eqs. 73 and 74 of the binodal a generic form for the solute free energy, $f_s(\phi) = S(\phi) - \chi\phi^2/2$, as a sum of the solution mixing entropy $S(\phi)$ and solute-solute attractive interaction energy $-\chi\phi^2/2$, where $\chi > 0$ is the attractive interaction energy (second virial coefficient), and ϕ is the solute volume fraction:

$$\frac{S(\phi_r) - S(\phi_p)}{\phi_r - \phi_p} = \frac{\partial S(\phi_r)}{\partial \phi_r} - \frac{\chi}{2}(\phi_r - \phi_p) - \frac{1}{2}\frac{\left(K^2\alpha^2 - p^2\beta^2\right)(M + \beta\phi_p)(\phi_r - \phi_p)}{\left((K + \beta\phi_r)(K + \beta\phi_p) + \frac{4\mu}{3}\left(K + \beta\bar{\phi}\right)\right)^2} \tag{88}$$

$$\frac{S(\phi_r) - S(\phi_p)}{\phi_r - \phi_p} = \frac{\partial S(\phi_p)}{\partial \phi_p} + \frac{\chi}{2}(\phi_r - \phi_p) + \frac{1}{2}\frac{\left(K^2\alpha^2 - p^2\beta^2\right)(M + \beta\phi_r)(\phi_r - \phi_p)}{\left((K + \beta\phi_r)(K + \beta\phi_p) + \frac{4\mu}{3}\left(K + \beta\bar{\phi}\right)\right)^2} \tag{89}$$

We adhere to the definition of the tie-lines as the locus of points in the $\chi - \bar{\phi}$ phase space, which determine the coexisting concentrations with equal chemical potential and osmotic pressure. Therefore, we invert the two equations above to find χ and $\bar{\phi}$ as a function of ϕ_r and ϕ_p. Since the mixing entropy is, by definition, independent of the interaction χ and, in the absence of long-range interaction between the solute (other than those mediated by the gel), is local and thus independent of $\bar{\phi}$, inverting the equations above results in:

$$\bar{\phi} = \frac{3}{4\mu\beta}\left(\sqrt{\frac{\beta\left(K^2\alpha^2 - p^2\beta^2\right)}{\left(4\frac{S(\phi_r)-S(\phi_p)}{\phi_r-\phi_p} - 2\left(\frac{\partial S(\phi_r)}{\partial\phi_r} + \frac{\partial S(\phi_p)}{\partial\phi_p}\right)\right)}(\phi_r - \phi_p)} - (K + \beta\phi_r)(K + \beta\phi_p)\right) - \frac{K}{\beta} \tag{90}$$

$$\chi = \frac{2}{\beta}\left(\frac{(M + \beta\phi_r)}{(\phi_r - \phi_p)^2}\frac{\partial S(\phi_r)}{\partial \phi_r} + \frac{(M + \beta\phi_p)}{(\phi_r - \phi_p)^2}\frac{\partial S(\phi_p)}{\partial \phi_p} - (2M + \beta(\phi_r + \phi_p))\frac{S(\phi_r) - S(\phi_p)}{(\phi_r - \phi_p)^3}\right) \tag{91}$$

These two equations show that for given values of ϕ_r and ϕ_p, there is only a single pair of $\bar{\phi}$ and χ values that solves Eqs. 73 and 74. Therefore, the notion of tie-lines is invalidated since the locus of points in the $\chi - \bar{\phi}$ phase space which determine the coexisting concentrations, is a single point in the phase space. This is in contrast to the fluid case (with no gel) where the coexisting concentrations are independent of th value of $\bar{\phi}$. Physically, this result can be attributed to the long-range interactions which couple the domain volumes to the coexisting concentrations within them. That way, for any domain size and geometry (which determines $\bar{\phi}$) there is a unique value of coexisting concentrations ϕ_r and ϕ_p. Notably, if $\beta = 0$, which accounts for the case where the solute does not affect the bulk modulus of the gel, $\bar{\phi}$ disappears from Eqs. 73 and 74, so they cannot be inverted to obtain $\bar{\phi}$. In this case, the binodal is independent of $\bar{\phi}$ and the tie-lines are restored.

References

[1] Akira Onuki. Ginzburg-landau approach to elastic effects in the phase separation of solids. *Journal of the Physical Society of Japan*, 58(9):3065–3068, 1989.

[2] Lev Davidovich Landau, Evgenii Mikhailovich Lifshitz, Arnold Markovich Kosevich, and Lev Petrovich Pitaevskii. *Theory of elasticity: volume 7*, volume 7. Elsevier, 1986.

[3] Samuel Safran. *Statistical thermodynamics of surfaces, interfaces, and membranes*. CRC Press, 2018.

[4] Anthony A Hyman, Christoph A Weber, and Frank Jülicher. Liquid-liquid phase separation in biology. *Annual review of cell and developmental biology*, 30:39–58, 2014.
